# Immunostimulatory Effect of Laminarin on RAW 264.7 Mouse Macrophages

**DOI:** 10.3390/molecules17055404

**Published:** 2012-05-08

**Authors:** Ji Young Lee, Young-Jin Kim, Hyun Joo Kim, Yoon-Sang Kim, Wansu Park

**Affiliations:** College of Korean Medicine, Gachon University, Seongnam 461-701, Korea; Email: oxygen1119@hanmail.net (J.Y.L.); godsentry@naver.com (Y.-J.K.); eternity0304@daum.net (H.J.K.)

**Keywords:** laminarin, macrophage, immunostimulatory, cytokine, nitric oxide, hydrogen peroxide, calcium, transcription factor

## Abstract

This study investigated the immunostimulatory effects of laminarin with respect to inflammatory mediators such as hydrogen peroxide, calcium, nitric oxide, various cytokines, transcription factors, and immune response gene in RAW 264.7 mouse macrophages. Laminarin did not reduce the cell proliferation of RAW 264.7 mouse macrophages at concentrations up to 500 µg/mL. Laminarin significantly increased the release of hydrogen peroxide, calcium, nitric oxide, monocyte chemotactic protein-1, vascular endothelial growth factor, leukemia inhibitory factor, and granulocyte-colony stimulating factor with enhancing expression of Signal Transducer and Activator of Transcription 1 (STAT1), STAT3, c-Jun, c-Fos, and cyclooxygenase-2 mRNA in RAW 264.7 cells. The results suggest that laminarin has immunostimulatory properties, strengthening immune reactions via the transcription factor pathway in macrophages.

## 1. Introduction

**Figure 1:**
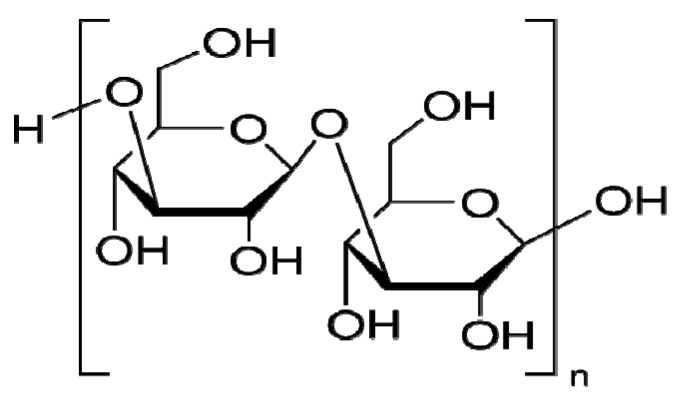
Structural formula of the Laminarin from *Laminaria digitata*.

Laminarin (Figure 1) is a storage glucan (a polysaccharide of glucose) found in brown algae. It is used as a carbohydrate food reserve in the same way that chrysolaminarin is used by phytoplankton. Laminarin is created by photosynthesis and is made up of β(1→3)-glucan with β(1→6)-linkages [1]. Botanical polysaccharides exhibit a number of beneficial therapeutic properties, and it is thought that the mechanisms involved in these effects are due to the modulation of innate immunity and, more specifically, macrophage function and macrophage hematopoiesis [2]. It was already reported that the water extract of *Aphanizomenon flos-aquae* (a freshwater species of blue-green algae) substantially increases mRNA levels of interleukin (IL)-1β and tumor necrosis factor (TNF)-α and enhances the DNA binding activity of nuclear factor-kappa B in human monocytic cells [3]. In addition, algae-derived polysaccharides such as Fucoidan, Immunon, Paramylo, and Immulina have also been reported to enhance the phagocytic and secretory activity of macrophages and induce the production of reactive oxygen species (ROS), nitric oxide (NO), and cytokines (TNF-α, IL-1, and IL-6) [2]. However, the immunomodulatory property of laminarin on macrophages is not yet fully reported.

In this study, we investigated the immunostimulatory effects of laminarin with respect to inflammatory mediators such as calcium, hydrogen peroxide (H_2_O_2_), NO, cytokines, transcription factors, and immune response gene in RAW 264.7 mouse macrophages.

## 2. Results and Discussion

**Figure 2:**
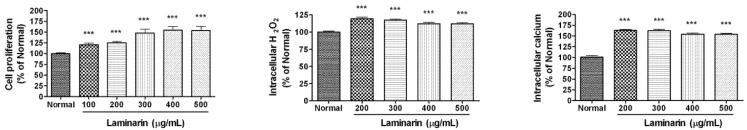
Effects of laminarin on the cell proliferation, calcium and, hydrogen peroxide production in RAW 264.7 mouse macrophages. Cells were incubated with laminarin for 24 h. Cell proliferation was evaluated by the MTT assay. Intracellular calcium was measured with Fluo-4 calcium assay. Intracellularhydrogen peroxide was measured with a dihydrorhodamine 123 assay. The normal group (Normal) was treated with medium only. Values are the mean + SEM of three independent experiments. *** *p* < 0.001 *vs.* Normal.

The proliferation of RAW 264.7 cells in the presence of laminarin at concentrations of 100, 200, 300, 400, and 500 µg/mL for 24 h were 120.8 ± 3.9%, 125.2 ± 3.01%, 147.9 ± 8.76%, 154.5 ± 8.6%, and 153.4 ± 10.09%, respectively (*p* < 0.001) of the normal group treated with medium only (Figure 2). Laminarin at concentrations of 200, 300, 400, and 500 µg/mL significantly increased intracellular calcium and H_2_O_2_ production in RAW 264.7 cells (*P* < 0.001) (Figure 2). These data suggest that laminarin might activate macrophages more safely than bacterial lipopolysaccharide (LPS) because LPS, which also increases intracellular calcium and H_2_O_2_ production in macrophages, also exerts cytotoxic effects on macrophages [4].

**Figure 3:**
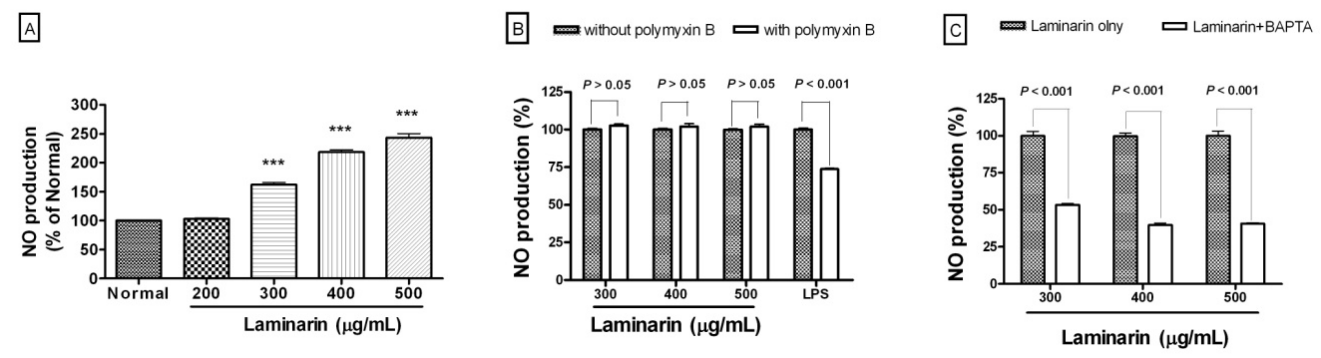
Effects of laminarin on NO production in RAW 264.7 mouse macrophages. After 24 h incubation with laminarin, NO productioninRAW 264.7 was measured by a Griess reaction assay. The normal group (Normal) was treated with media only. Polymyxin B (0.5 µg/mL), an inhibitor of lipopolysaccharide (LPS; 1 µg/mL), did not show the significant effect on laminarin-induced NO production (**A** and **B**), whereas laminarin-induced NO production was significantly inhibited by intracellular calcium chelator, BAPTA-AM (BAPTA; 10 µM) (**C**). Values are the mean + SEM of three independent experiments. *** *p* < 0.001 *vs.* Normal.

Laminarin significantly increased NO production in RAW 264.7 cells at concentrations of 300, 400, and 500 µg/mL (*p* < 0.001) (Figure 3A). Polymyxin B (0.5 µg/mL), an inhibitor of LPS, did not show the significant effect on laminarin-induced NO production (Figure 3B). The intracellular calcium chelator, 1,2-bis-(o-aminophenoxy)-ethane-*N,N-N′,N′-* tetraacetic acid tetraacetoxymethyl ester (BAPTA-AM) applied at a concentration of 10 µM significantly (*p* < 0.001) inhibited NO production of RAW 264.7 cells induced by laminarin (Figure 3C). These results indicate that laminarin activates macrophages to release inflammatory mediators such as NO via a mechanism involving, at least in part, calcium signaling.

**Figure 4:**
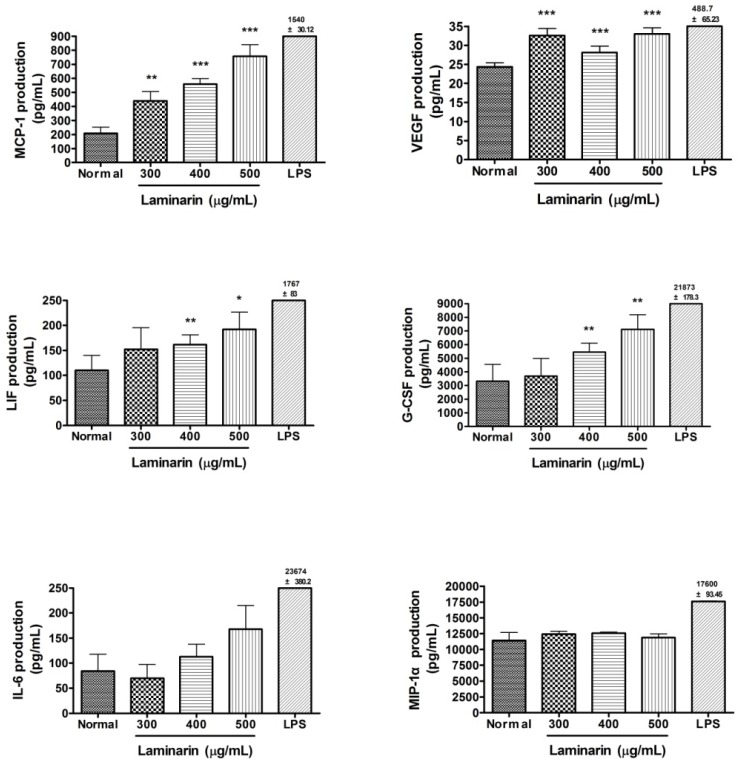
Effects of laminarin on cytokine production (MCP-1, VEGF, LIF, G-CSF, IL-6, and MIP-1α) in RAW 264.7 mouse macrophages. After 24 h incubation with laminarin, fluorescence intensity of each cytokine in the culture medium was measured by Multiplex bead-based cytokine assay. The normal group (Normal) was treated with medium only. Lipopolysaccharide (LPS; 1 µg/mL) was used as a positive control. Values are the mean + SEM of three independent experiments. * *p* < 0.05 *vs.* Normal; ** *p* < 0.01; *** *p* < 0.001.

The effects of laminarin on production of cytokines in RAW 264.7 cells are shown in Figure 4. RAW 264.7 cells were incubated with laminarin at concentrations of 300, 400, and 500 µg/mL for 24 h. After 24 h incubation, laminarin significantly increased the production of monocyte chemotactic protein-1 (MCP-1) and vascular endothelial growth factor (VEGF) at concentrations of 300, 400, and 500 µg/mL (*p* < 0.01). Also, laminarin significantly increased the production of leukemia inhibitory factor (LIF) and granulocyte-colony stimulating factor (G-CSF) at concentrations of 400 and 500 µg/mL (*p* < 0.05). But the production of IL-6 and MIP-1α was not significantly increased by laminarin preparation. Because LPS explosively increases the production of IL-6 and MIP-1α in macrophages, these data mean that the immunostimulatory effect of laminarin on macrophages is different from that of LPS.

Immunostimulated macrophages produce large amounts of ROS, such as H_2_O_2_, which causes oxidative stress, resulting in macrophage reprogramming with a transient increase of intracellular calcium via the lipid membrane dissociation [5]. This increased cytosolic calcium, in turn, activates calcium-dependent transcription factors (TFs) including Signal Transducer and Activator of Transcription (STAT) 1, STAT3, and Activator protein-1 (AP-1; a heterodimeric protein composed of c-Fos and c-Jun), subsequently increasing the transcription of proinflammatory target genes [6,7,8].

**Figure 5:**
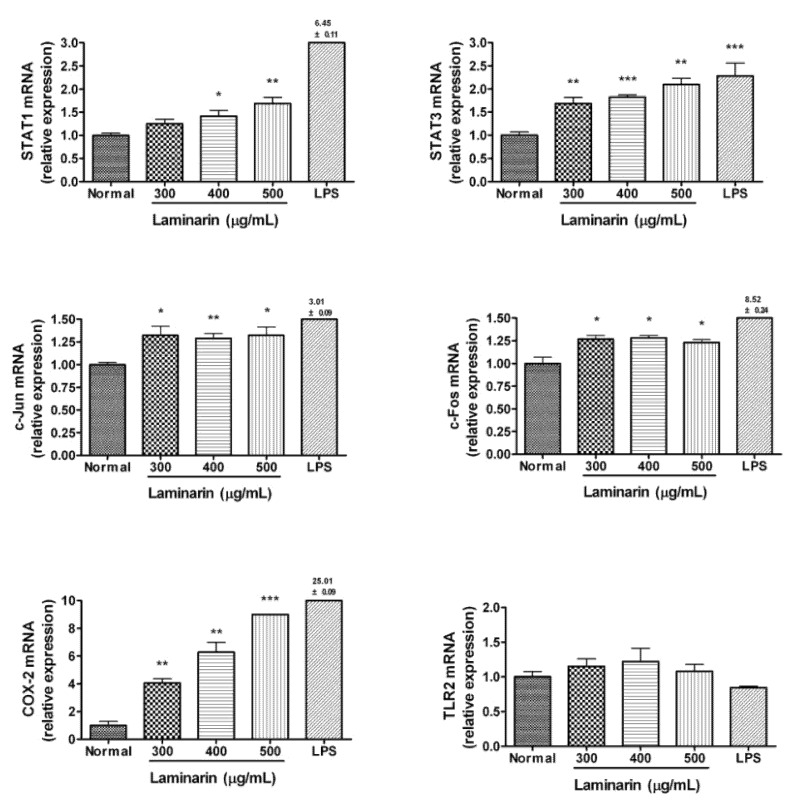
Effects of laminarin on mRNA expression of STAT1, STAT3, c-Jun, c-Fos, COX-2, and TLR2 in RAW 264.7 mouse macrophages. After 24 h incubation with laminarin, immune-related gene mRNA in cells was measured by a Quantitative bead-based multiplex gene assay. The normal group (Normal) was treated with medium only. Lipopolysaccharide (LPS; 1 µg/mL) was used as a positive control. Values are the mean + SEM of the three independent experiments. * *p* < 0.05 *vs.* Normal; ** *p* < 0.01; *** *p* < 0.001.

The effects of laminarin on mRNA expression of immune-related genes in RAW 264.7 cells are shown in Figure 5. RAW 264.7 cells were incubated with laminarin at concentrations of 300, 400, and 500 µg/mL for 24 h. After 24 h incubation, all concentrations of laminarin significantly increased mRNA expression of STAT3, c-Jun, c-Fos and cyclooxygenase (COX)-2 (*p* < 0.05). Laminarin also significantly increased mRNA expression of STAT1 at concentrations of 400 and 500 µg/mL (*p* < 0.05), but laminarin did not show a significant effect on toll-like receptor 2 mRNA expression.

In the current study, laminarin enhanced the mRNA expression of STAT1, STAT3, c-Jun, c-Fos, and COX-2 with increasing calcium and H_2_O_2_ in RAW 264.7 macrophages. These results suggest that the activation of macrophages by laminarin might be achieved via the transcription factor pathway that includes oxidative stress, calcium, STAT1, STAT3, AP-1, and COX-2.

Recently, botanical polysaccharides have attracted much attention in the biomedical field because they enhance macrophage immune responses, leading to immunomodulation, anti-tumor activity, wound-healing and other therapeutic effects [2]. Thus, the present study suggests that laminarin has the potential to be as useful therapeutic agent with immunostimulatory, anti-tumor, and wound-healing properties.

## 3. Experimental

### 3.1. Reagents

Dulbecco’s Modified Eagle’s Medium (DMEM), heat-inactivated fetal bovine serum (FBS), penicillin and streptomycin, phosphate-buffered saline (PBS, pH 7.4), and other tissue culture reagents were purchased from Gibco BRL (Grand Island, NY, USA). 3-(4,5-Dimethyl-2-thiazolyl)-2,5-diphenyltetrazolium bromide (MTT), LPS, Griess reagent, BAPTA-AM, dihydrorhodamine 123 (DHR), polymyxin B, and all other chemicals were purchased from Sigma-Aldrich (St. Louis, MO, USA). The multiplex bead-based cytokine assay kits used for the determination of cytokine concentration were purchased from Bio-Rad (Hercules, CA, USA) and Millipore (Billerica, MA, USA). QuantiGene Plex 2.0 assay kit was purchased from Panomics (Redwood City, CA, USA). The Fluo-4 calcium assay kit was purchased from Molecular Probes (Eugene, OR, USA).

### 3.2. Cell Culture and Proliferation

RAW 264.7 mouse macrophages were obtained from the Korea Cell Line Bank (Seoul, Korea). RAW 264.7 were cultured in DMEM supplemented with 10% FBS containing 100 U/mL of penicillin and 100 µg/mL of streptomycin at 37 °C in a 5% CO_2_ humidified incubator. After RAW 264.7 cells were seeded in wells of a 96-well plate, laminarin was added to the culture medium and incubation was continued for 24 h at 37 °C. Cell proliferation was assessed using the MTT assay.

### 3.3. Intracellular Calcium Assay

After RAW 264.7 cells were seeded in wells of 96-well plates, laminarin was added to the culture medium, and incubation was carried out for 24 h at 37 °C. Thereafter, the medium was removed and cells were incubated with 100 µL of the Fluo-4 dye loading solution for 30 min at 37 °C, then at room temperature for an additional 30 min. After incubation, the fluorescence intensity of each well was determined using a spectrofluorometer (Dynex, West Sussex, UK) with excitation filter 485 nm and emission filter 535 nm.

### 3.4. H_2_O_2_ Assay

The intracellular production of H_2_O_2_ was measured with DHR, as described previously in detail [9]. During the cellular production of ROS, the nonfluorescent DHR was oxidized by H_2_O_2_ and irreversibly converted to the green fluorescent compound rhodamine 123 (R123). R123 was membrane-impermeable and accumulated in the cells. An aliquot of DHR (to produce a concentration of 10 µM in each well) was added to each 96-well plate and preincubated for 30 min at 37 °C. Thereafter, the medium was removed and RAW 264.7 cells were incubated with laminarin for 24 h at 37 °C. After incubation, fluorescence intensities of each well were analyzed by spectrofluorometer (Dynex) with excitation filter 485 nm and emission filter 535 nm.

### 3.5. Quantification of NO Production

NO concentration in culture medium was determined by the Griess reaction assay [10]. Specifically, 100 µL of supernatant from each well was mixed with 100 µL of Griess reagent in wells of a 96-well plate. After an incubation of 15 min at room temperature, the optical density was determined at 540 nm with a microplate reader (Bio-Rad).

### 3.6. Multiplex Bead-Based Cytokine Assay

Cytokines released from RAW 264.7 macrophages treated with laminarin were measured in cell culture supernatants using a Luminex assay based on xMAP technology. This assay was performed with Bio-Plex cytokine assay kits (Bio-Rad), Milliplex kits (Millipore) and Bio-Plex 200 suspension array system (Bio-Rad) as described previously [10,11,12]. Standard curves for each cytokine were generated using the kit-supplied reference cytokine samples. Production of the following cytokines was assessed: MCP-1, VEGF, LIF, G-CSF, IL-6, and MIP-1α.

### 3.7. Direct Quantification of Multiple RNA Targets

At the end of 24 h incubation with laminarin, RAW 264.7 cells were lysed. To simultaneously quantify multiple RNA targets directly from cell lysate, QuantiGene Plex 2.0 Reagent System (Panomics) based on branched DNA signal amplification technology with xMAP beads was used according to manufacturer’s instructions [11,12]. The mRNA expressions of STAT1, STAT3, c-Jun, c-Fos, COX-2, and TLR2 were determined with housekeeping gene TATA-binding protein (TBP).

### 3.8. Statistical Analysis

The results shown were from at least three independent experiments. Significant differences were examined by analysis of variance, followed by Dunnet’s post hoc test for multiple comparisons, or with Student’s *t*-test for two group comparisons. Significance was assumed at *p* < 0.05.

## 4. Conclusions

The present study has demonstrated that laminarin significantly increased the release of calcium, H_2_O_2_, NO, MCP-1, VEGF, LIF, and G-CSF with enhanced expression of STAT1, STAT3, c-Jun, c-Fos, and COX-2 in RAW 264.7 cells. These results suggest that laminarin has immunostimulatory properties, and strengthens immune reactions via the TF pathway in macrophages. Further studies are needed to verify the precise mechanism regulating the immunostimulatory activities of laminarin.
